# Improving Health of People With Multiple Sclerosis From a Multicenter Randomized Controlled Study in Parallel Groups: Preliminary Results on the Efficacy of a Mindfulness Intervention and Intention Implementation Associated With a Physical Activity Program

**DOI:** 10.3389/fpsyg.2021.767784

**Published:** 2021-12-24

**Authors:** Eya Torkhani, Emilie Dematte, Jean Slawinski, Antonia Csillik, Marie-Claire Gay, Djamel Bensmaïl, Olivier Heinzlef, Giovanni de Marco

**Affiliations:** ^1^Laboratoire LINP2, Université Paris Nanterre, UPL, Nanterre, France; ^2^French National Institute of Sport, Expertise and Performance, Sport, Expertise and Performance Laboratory, Paris, France; ^3^iMSpire (International Multiple Sclerosis Partnership in Research) Special Interest Group, Paris Nanterre University, Nanterre, France; ^4^Department of Psychology, Université Paris Nanterre, Nanterre, France; ^5^Department of Physical and Rehabilitation Medicine, Raymond Poincaré Hospital – APHP Paris Saclay, Garches, France; ^6^UMR 1179 INSERM-UVSQ, Neuromuscular Handicap – University of Versailles, Montigny-le-Bretonneux, France; ^7^CHI de Poissy-St Germain, Conflans-Sainte-Honorine, France

**Keywords:** multiple sclerosis, positive psychology interventions, mindfulness, implementation intention, physical activity, quality of life

## Abstract

**Objectives:** The objective of this study is to investigate the efficacy of psychological Interventions – Mindfulness or Implementation Intention – associated with a Physical Activity program, delivered via internet, in reducing Multiple Sclerosis symptoms.

**Method:** Thirty-five adults were randomly assigned to one of the three groups: a Mindfulness-Based Intervention group (*N* = 12), Implementation Intention group (*N* = 11), and a Control Group (*N* = 12). All the groups received the same Physical Activity program. The Mindfulness condition group received daily training in the form of pre-recorded sessions while the Implementation group elaborated their specific plans once a week. Mobility, fatigue, and the impact of the disease on the patient’s life were measured. Two measurement times are carried out in pre-post intervention, at baseline and after eight weeks.

**Results:** Overall, after 8 weeks intervention, results show that there was a significant increase in Walking distance in the three groups. In addition, the within-group analysis showed a statistically significant improvement between pre and post intervention on the physical component of the Disease Impact scale in the Implementation Intention group (*p* = 0.023) with large effect size, in the Mindfulness-Based Intervention group (*p* = 0.008) with a medium effect size and in the control group (*p* = 0.028) with small effect size. In the Implementation Intention group, all physical, psychosocial and cognitive Fatigue Impact subscales scores decreased significantly (*p* = 0.022, *p* = 0.023, and *p* = 0.012, respectively) and the physical component was statistically and negatively correlated (*r* = *−0.745; p* = *0.008*) when Implementation Intention group practice a mild to moderate physical activity. In the Mindfulness-Based Intervention group, the physical component (MFIS) showed a statistically significant improvement (*p* = 0.028) but no correlation with moderate-to-vigorous physical activity (MVPA); the control group outcomes did not reveal any significant change.

**Conclusion:** The results of this study are very encouraging and show the feasibility of Mindfulness interventions associated with physical activity to improve the health of people with MS. Further study should assess Mindfulness interventions tailored to MS condition and using both hedonic and eudemonic measures of happiness.

## Introduction

Multiple sclerosis (MS) is an autoimmune disease of the central nervous system (CNS) with physical, emotional, and cognitive symptoms that substantially reduce participants’ overall quality of life (Amato et al., 2001; Benedict et al., 2005). Patients typically present with a relapsing-remitting course of the disease that is characterized by periodic exacerbations, followed by recovery and stretches of relative stability (Lublin and Reingold, 1996; Ajzen, 2013). The spectrum of the disease itself generates a range of manifestations and severities which mainly concern the central nervous system (Hauser and Oksenberg, 2006; Trapp and Nave, 2008).

Physical Activity (PA) may be interesting in people with MS. Initially, PA was discouraged in people with MS due to concerns about fatigue and temperature sensitivity (Petajan et al., 1996; Dalgas et al., 2008). However, a number of clinical investigators revealed that exercise is, in fact, beneficial for MS patients’ as it may slow down the disease process and ensure the stability and management of its symptoms (Hessen et al., 2006; Motl and Gosney, 2008; Khan et al., 2014) as long as it is designed to “activate working muscles, but avoid overload that results in conduction block” (Petajan and White, 1999). Physical activity is considered as a possible behavioral approach for managing MS symptoms and secondary health problems associated with an inactive lifestyle (Petajan et al., 1996; Dalgas et al., 2008; Motl and Gosney, 2008; Asano et al., 2009; Motl and Pilutti, 2012; Carter et al., 2014; Sá, 2014; Turner et al., 2015).

Recent MRI studies carried out on Murine animal models (Jensen et al., 2018) and human models (Negaresh et al., 2019) tend to demonstrate that PA practice would enhance axon remyelination. Moreover, PA participation may increase general self-efficacy (Kasser, 2009). The latter affects positively the psychological, psychosocial and cognitive outcomes (Barnwell and Kavanagh, 1997; Dlugonski et al., 2012; Ellis and Motl, 2013; Hughes et al., 2015). And yet this population is largely sedentary and inactive compared with the general population (Motl et al., 2006), even if they are mildly affected by MS (Ng and Kent-Braun, 1997; Stuifbergen and Roberts, 1997) and 80% of them do not meet recommended levels of moderate-to-vigorous physical activity (MVPA) (Motl, 2014). Research efforts have implicated several physical, mental and environmental barriers limited their participation in PA (Crayton et al., 2004; Dodd et al., 2006; Kerdoncuff et al., 2006; Aminian et al., 2019). This reality highlights the importance of developing behavior change interventions aiming at helping the patients to initiate and sustain his practice.

Many psychological interventions used with people with chronic conditions such as MS propose a deficit-oriented model in which psychotherapists assess and treat psychopathology and disease associated symptoms, without exploring and developing patients’ resources, strengths and positive emotions. Few interventions have explicitly attended to the positive resources of people with MS. The field of Positive Psychology (PP) proposes several empirically based interventions that focus on clients’ positive attributes, strengths, and positive emotions (Csillik, 2015). Positive psychology interventions (PPIs) are treatment methods or intentional activities that aim to cultivate positive feelings, behaviors, or cognitions. These forms of interventions (e.g., positive psychotherapy, well-being therapy, mindfulness based-interventions, etc.) consist mainly of structured forms of volitional activities (Sin and Lyubomirsky, 2009). Positive psychology interventions refer to systematic approaches to overcome everyday challenges and life’s difficulties and psychopathology by using clients’ strengths and by cultivating positive emotions. The aim of the PPIs is thus both to relieve suffering and psychopathology and increase well-being. PPIs essentially involve the reeducation of attention and memory (Rashid, 2009), by practicing mindfulness, kindness and forgiveness, expressing gratitude and using personal strengths etc. The PPIs have shown their effectiveness in increasing well-being, both in the general population and clinical samples (Sin and Lyubomirsky, 2009; Bolier et al., 2013). To date, to our knowledge no published study assessed the efficacy of positive psychology interventions associated with physical activity in people with MS. One of the first positive psychology interventions, Mindfulness or Meditation, “a family of self-regulation practices that focus on training attention and awareness in order to bring mental processes under greater voluntary control” (Walsh and Shapiro, 2006, p. 228), has shown encouraging results in relation to improving physical and mental health problems and increasing well-being (Brand et al., 2012; Davidson and McEwen, 2012; Willekens et al., 2018). One of the most popular meditative practices nowadays is mindfulness meditation, which cultivates a state of non-judgmental awareness of the present moment. Mindfulness is a practice that originate from ancient Buddhist meditation techniques, but have since been empirically tested, manualized, and adapted for use in a diverse range of clinical settings (Kabat-Zinn, 2003) so that it is now part of mainstream psychotherapeutic interventions (Williams and Kabat-Zinn, 2011).

In the context of MS, Mindfulness may help people to become conscious of repetitive perceptions about the past or disturbance thoughts about the unknown future without necessarily engaging or pursuing them. Nydahl (2012) has shown that the mindfulness training program has a positive effect on MS patients. When studying the idea that emotions are transitory and constantly changing, the intervention generates confidence in their ability to shape their lives. This training can minimize the emotional and psychological impact of chronically ill patients and help improve clinical conditions, such as reducing fatigue (Trojan et al., 2007; Bol et al., 2009). Furthermore, Mindfulness might be a way of dealing with varying common visible (mainly physical) and hidden MS symptoms at the same time (Senders et al., 2014). Indeed, Mindful people with MS reported reduced emotional distress (Hart et al., 2005; Mohr et al., 2007; Van Kessel et al., 2008; Bogosian et al., 2015), improved balance (Mills and Allen, 2000; Burschka et al., 2014), reduced pain (Tavee et al., 2011), improved quality of life and reduced depression and fatigue (Grossman et al., 2010). Mindful based-interventions (MBIs) might also positively affect accuracy of perception, acceptance of intractable health-related changes, realistic sense of control, and appreciation of available life experiences.

Implementation intention (II) (or if-then plan) was introduced by Gollwitzer (1999). The concept of II is rooted in the theory of self-regulation (Leventhal et al., 1998). The latter is so distinguished from the concept of goal intention. Goal intention specify what one wants to achieve (i.g., “I intend to achieve X!”). However, using this strategy people generally fail to translate their intention into action as research has perceived a pervasive gap between the intention formulated and the desired behavior (Ajzen, 1991). II, defined as a self-regulatory strategy in which a psychological link is established between a predefined future situation and a desired goal-oriented response (Gollwitzer, 1993, 1999), involves specifying the behavior one will perform in the service of the goal and the situational context in which one will enact it (e.g., “If I encounter situation X, then I will initiate action Y!”) (Sheeran et al., 2005). The success of II is assumed to be based on two processes. First, by making the critical situation highly accessible (i.g., the ‘if’ component), and second, by leading to the automatic initiation of the specified behavior (the ‘then’ component) (Webb and Sheeran, 2004). This strategy is centered on designating anteriorly, rigorously, and explicitly where, when, and how a goal-directed response will be executed, resulting in facilitated goal attainment (Gollwitzer and Sheeran, 2006; Cohen et al., 2007; De Vet et al., 2011). The II strategy have shown its effectiveness in promoting goal achievement (Sheeran and Orbell, 2000), with an effect size on goal achievement for health-related behavioral change estimated as medium to large (Gollwitzer and Sheeran, 2006). Numerous interventions have used II strategy in different fields and among varied populations even in people with chronic conditions such as cancer and diabetes (Verplanken and Faes, 1999; Sheeran and Orbell, 2000; White et al., 2012; Bélanger-Gravel et al., 2013). Findings were promising and positive, and all support the fact that II is a powerful strategy designed to facilitate goal attainment. For people with MS, If-then plans are advantageous in terms of planification and programming of action. In fact, patients’ knowledge, skill, walking speed, quality of life and confidence in managing their health or chronic condition were improved after an II intervention conducted by (Kersten et al., 2015). Also, Electroencephalography (EEG) and functional Magnetic Resonance Imaging (fMRI) studies have shown that II strategy modulates our perceptions and produces less activity in brain regions involved in effort-motivated behavioral control (Wieber et al., 2015).

The aim of our clinical study was to explore the efficacy of two types of psychological interventions – Mindfulness and Implementation of Intention (II) – delivered via internet among people with MS when associated and compared to a physical activity program. We investigated the hypothesis that combining a PA program with Mindfulness or Implementation Intention may be more beneficial than PA alone in decreasing fatigue and improving mobility and quality of life in people with MS.

## Materials and Methods

### Design

This study is a randomized controlled pilot trial of a brief intervention combining Physical activity, Mindfulness training and Implementation intention to decrease fatigue and improve mobility among people with MS. The primary outcomes, fatigue, mobility and quality of life, were examined at baseline prior to randomization, and then 8-weeks after the intervention (following the online intervention).

### Participants

Participants were recruited from two hospitals in the Parisian region, in France receiving outpatient services for MS at a regional medical center. Thirty-five patients (*N* = 28 Females) were recruited and gave their written consent to participate to this study. Inclusion criteria for the study included (1) a diagnosis of MS, (2) not currently engaged in rehabilitation, therapy or PA program and able to undertake PA without risk-factors (3) aged between 18 and 65 (4) able to give informed consent and to understand French. We excluded those who were receiving drug treatment for fatigue started less than three months ago or acute hospital or nursing home, those who were having psychiatric disorders. All participants had a definite diagnosis of MS (diagnosed 12 months previous and relapse free in previous 90 days). Of the 35 patients, 25 were diagnosed with relapsing-remitting MS: 7 in II group; 8 in MBI group and 10 in the CG (control group); three were diagnosed with primary progressive MS: 1 in II group 1 in MBI group and 1 in the CG; seven were diagnosed with secondary progressive MS: 3 in II group; 3 in MBI group and 1 in the CG. They had a mean score of disability of [3.24, Expanded Disability Status Scale (EDSS)<5.5] and were able to consult at the outpatient clinic. Patients were persevering in Physical Activity. Only, three of them dropped out the PA program before the end.

### Procedure

This clinical and multicentric trial took place from July 2017 to January 2020, in two hospitals in the Parisian Region, France (Hospitals – CHI Poissy St Germain-en-Laye and R. Poincaré, Garches). The procedures for this study were approved by research ethics committees (CPP) «Ile de France XI » (ID: 2016-A00537-44), authorized from The National Agency for the Safety of Medicines and Health (ASNM) and registered under the identification number ID: NCT03785483. For this study, individuals were approached by the study coordinator and asked if they would be willing to participate in it. All participants provided written informed consent and underwent a neurological exam by two physicians who provided EDSS scores considered as a clinical measure of disability (Goodin, 1998). Participants initially completed demographic and clinical evaluation (including age range, gender, diagnoses and mobility). Transmission and reception of the adhesion to the psychological and motivational interventions and assessment were carried out due to TailorBuilder tool^1^. TailorBuilder is an online tool used to develop programs for creating and conducting web-based questionnaires and designing tailor-made programs (Ruffault et al., 2016a,b, 2019). Such connected tools guarantee traceability and securing the data collected. They are also beneficial since they permit to collect subject’s subjective experiences as close as possible to the event and provides information regards their behavior moment by moment. All measurements were carried out at two phases of the study T1 (baseline) and T2 (eight weeks after randomization). A specialized trainer in adapted physical activity delivered experimental interventions and the telephone calls. Outcomes and information collected from the telephone calls sessions were recorded on a data sheet, different for each participant.

Participants completed an intake screening online questionnaire. Baseline demographic information (e.g., age, gender, education) and disease information (e.g., years with MS, mobility disability, fatigue, cognitive functioning) were obtained at intake. Following completion of baseline, individuals were individually and aleatory affected to one of the three groups. Randomization list was then delivered to the trainer. Prior to assignment, treatment condition was unknown to the project coordinator to ensure concealment of allocation.

Individuals participated in either a combination of PA and daily Mindfulness or a combination of PA and Implementation Intention. Mindfulness-Based Intervention group (MBI) (*n* = 12) received daily Mindfulness training in the form of prerecorded sessions (6 sessions of 10 minutes each equal to one hour/day) using TailorBuilder (TB) application, aiming at developing awareness of emotions and sensations. Mindfulness practice took place individually at home six times per week, without any assistance. Implementation Intention group (II) (*n* = 11) was asked to establish a concrete if-then plan (the ‘if’ component followed by the ‘then’ component) once a week and share them through the TB application. These combinations were intended to decrease fatigue and improve mobility among people with MS. PA consists of the combination of four activities per week: 40 minutes of walking, 40 minutes of muscle building, 20 minutes of stretching and 20 minutes of coordination and balance, with a frequency of 2 hours weekly. The PA intervention sessions were scheduled at a time and place of the participant’s choosing and connections to the site checked. Practicing sessions were conducted under the supervision of a physical and sports activity trainer specializing in sports training. Participants were randomly assigned to one of the three experimental groups: MBI group, II group or Control Group (CG) (*n* = 12). Participants in the control group were not guided to develop if-then plans and they did not receive any mindfulness training, however, they received the same PA program over a period of eight weeks. All participants were analyzed in the group to which they were designed and ignored the existence of the other study groups. According to the recommendations of McCoy (2017), concerning the randomized controlled trials and the assess of the psychological interventions efficacy all the patients of the study were analyzed. The missing values in session 2 (one MBI and two CG) were replaced by the group average.

Participant’s clinical characteristics are provided in Table 1.

**TABLE 1:** Clinical characteristics of the 35 participants (Median ± Interquartile Range).

	Group		
	II(*n* = 11)	MBI(*n* = 12)	CG(*n* = 12)
6MWT (meter)	435.00[287.50–477.50]	429.50[264.75–476.25]	458.50[353.25–515.00]
MFIS (Cognitive component)	19.00[13.00–23.00]	16.00[4.50–25.75]	17.50[11.50–22.00]
MFIS (physical component)	23.00[21.00–28.00]	20.50[14.75–26.75]	22.50[16.75–23.50]
MFIS (Psychosocial component)	4.00[2.50–6.00]	5.00[1.50–2.25]	4.00[2.75–4.25]
MSIS-29_(physical component)	47.00[41.50–52.00]	43.00[30.75–54.00]	48.50[32.00–57.50]
MSIS-29_(psychological component)	25.00[20.00–26.00]	25.00[13.75–33.75]	22.00[17.75–26.25]

*Values presented are median [interquartile range] for each characteristic. II = Implementation Intention, MBI = Mindfulness Based Intervention, CG = Control Group, 6MWT = 6 Min Walking Test, MFIS = Modified Fatigue Impact Scale, MSIS-29 = Multiple Sclerosis Impact Scale-29.*

### Instruments

#### Demographic Information

Age, gender, marital status (currently married vs. all other) and education level (in years) were all obtained from single-item queries.

After medical history screening, mobility, fatigue, quality of life and the impact of the disease on the patient’s life were measured by 6 min Walking Test (6MWT) (Guyatt et al., 1985) Modified Fatigue Impact Scale (MFIS) (Téllez et al., 2005), Health-related quality of life measured with EuroQoL (EQ5D-3L) (Goodwin et al., 2019) and Multiple Sclerosis Impact Scale (MSIS-29) (Hobart, 2001), respectively.

#### Expanded Disability Status Scale

Expanded Disability Status Scale (EDSS) (Kurtzke, 1983), a widely used and extensively validated physician-rating tool that estimates disease-related impairment in persons with MS was used.

#### Modified Fatigue Impact Scale

Fatigue was measured with the Modified Fatigue Impact Scale (MFIS). The original MFIS assesses the impact of fatigue in physical, cognitive, and psychosocial domains (Fischer et al., 1999). Participants rate the frequency of impact upon daily life in the past 4 weeks using values ranging from 0 (never) to 4 (almost always). The Modified Fatigue Impact Scale was translated and culturally adapted into French. The psychometric properties of this new instrument, called EMIF-SEP composed of 40 items are good. Four dimensions of this scale (cognitive, physical, social role and psychological) were identified by factor analysis, each with a high internal consistency (Cronbach’s alpha = 0.94, higher than 0.80 in APA standard). The test-retest reproducibility was very satisfactory (ICC = 0.93); intra-class correlation coefficients were all above 0.70. EMIF-SEP is the first scale for assessing MS-related fatigue which has been adapted to French-speaking patients.

#### The Multiple Sclerosis Impact Scale

The Multiple Sclerosis Impact Scale (MSIS-29) is a measure of the physical and psychological impact of multiple sclerosis from the patients’ perspective. The MSIS-29 satisfied all psychometric criteria. Item test-re-test reliability was high (*r* = 0.65–0.90) and scale scores could be generated for >98% of respondents. Item descriptive statistics, item convergent and discriminant validity, and factor analysis indicated that it was legitimate to generate scores for MSIS-29 scales by summing items. MSIS-29 scales showed good variability, small floor and ceiling effects, high internal consistency (Cronbach’s alpha = 0.93) and high test-re-test reproducibility (ICC > 0.90).

#### The 6-min Walk Test

Originally developed to assess disability in patients with chronic obstructive lung disease, the 6-min walk test (6MWT) has since been extensively studied and used with older persons and patients, also in people with MS.

Initially, developed to assess disability in people suffering from chronic obstructive pulmonary disease, the 6-min test (6MWT) has still been widely studied in the elderly, as well as in people with MS (Eng et al., 2002; Newman et al., 2003). The 6MWT has the advantage of potentially being able to demonstrate motor fatigue in people with MS, that is, slowing of ambulation at the end of the 6-min test. The 6MWT is a feasible and reproducible measure in MS (Goldman et al., 2008), and it has high reliability (ICC: 0.95–0.99) in people with MS even after a single repetition of the test (Fry and Pfalzer, 2006) in addition, it is sensitive to changes in deteriorating status among people with MS (Paltamaa et al., 2008).

#### EQ-5D-3L

The EuroQoL (EQ-5D-3L) is often used as a quantitative measure of health outcome that reflects the patient’s own judgment (Szende et al., 2014). It has five dimensions (mobility, self-care, usual activities, pain/discomfort, anxiety/depression). Each dimension has 3 levels: no problems, some problems, and extreme problems. The patient is asked to indicate his/her health state by ticking the box next to the most appropriate statement in each of the five dimensions. The EQ-5D is widely used in economic evaluations, recommend as the preferred measure of health outcomes for cost effectiveness analyses (Jones et al., 2013).

#### Accelerometry

The Actigraph GT3X accelerometer (Health One Technology, For Walten Beach, Fla.) was used to measure participants’ PA practice at T1 and T2 in their daily lives. Data were collected during the day for 7 consecutive days, before and immediately after eight weeks of the PA program. The GT3X was worn at the waist, a suitable fit for measuring the amount of PA in people with MS (Weikert et al., 2010). Participants were shown how to wear the device. In addition, detailed written instructions were delivered, containing all the necessary information on wearing the equipment provided. Participants were instructed to wear it constantly except for sleeping, swimming, and when washing. The ActiGraph software was used to measure the time spent in Moderate Vigorous Physical Activity (MVPA) (min/day) according to the cut points determined by Sandroff’s study (Sandroff et al., 2014) and validated for people with MS regarding their EDSS score. Accelerometer recorded wear time was examined and only valid days (≥ 10 h of wear time without periods of continuous zeros exceeding 60 min indicative of non-compliance) were used in the analysis.

### Interventions

#### Physical Activity Program

The three groups received the same PA program, which was controlled for contraindications and delivered by a neurologist (OH) at Poissy CHI Hospital (France) and a specialist in physical medicine and rehabilitation (DB) at Raymond-Poincaré Hôpital – Garches (France). The PA program was manualized and available as a booklet. This program initiated after the primary actimetry measurement and maintained for 8 consecutive weeks with a frequency of 2 h weekly. Practicing sessions took place at home under the supervision of a physical and sports activity trainer specializing in sports training. Patients combined four activities per week: 40 min of walking, 40 min of muscle building, 20 min of stretching and 20 min of coordination and balance. Progression in terms of duration and intensity were proposed. First, an increase in exercises duration and then an increase in intensity (associated with a decrease in duration if necessary). This progression respects the classic rules of applied in sport science. The exercises aimed to induce mild to moderate fatigue and were stopped if they engender excessive fatigue which was based on the feeling of the patient and on his own evaluation.

#### Mindfulness Intervention

MBI participants received daily mindfulness training in the form of prerecorded sessions (6 sessions of 10 min each equal to 1 h/day) using TailorBuilder application, aiming at developing awareness of emotions and sensations. Mindfulness practice took place individually at home without any assistance six times per week. Participants were asked to listen to the prerecorded sessions and to follow the instructions (e.g., Breathing rhythm, number of steps during walking exercises, speed of execution of the movement). Participants also received a weekly telephone call lasted up to 15 min, during which a detailed report concerning the session(s) was reviewed and adapted if required. Participants were asked to describe their sessions and to specify the number and duration of each one. Moreover, they were asked to describe the sensations and to evoke any event or situation that made the exercise easier or even harder to perform.

#### Implementation Intention

Intention Implementation Participants were asked to develop a concrete if-then plan (the “if” component followed by the “then” component) once a week and share them through TailorBuilder app. Subjects were asked to specify the conditions (e.g., *when*, *where*, *how, what*) in which the behavior is expected to be performed. Participants were able to modify their plans depending on their physical condition and availability. These plans were verified and approved by the trainer. Implementation Intention assessment was carried out weekly via internet questionnaire. Participants also received a weekly telephone call lasted up to 15 min, during which a detailed report concerning the session(s) performed was reviewed and refined if required. Participants were asked to specify the number and duration of each session, sensations, facilitators, and barriers encountered to sustain their practice.

### Statistical Analyses

We used non-parametric tests due to the small sample sizes which deviate from the normality and equal variance assumptions. First, the Kruskal-Wallis test was used to analyze the homogeneity of groups in relation to sociodemographic characteristics. Second, comparisons between pre and post intervention were analyzed in each group using the Wilcoxon signed-ranked *t*-test for within-group differences, and effect sizes were given by the matched rank biserial correlation (Kerby, 2014), interpreted as Person’s *r*. We used Spearman’s rank correlation test, corrected for multiple comparisons, to assess the relationships between MVPA and fatigue. Statistical analysis was carried out using JASP version 0.14.1 software, with a significance level of *p* < 0.05.

## Results

### Demographic Analysis and Quality of Life

Most of our study sample was female (*n* = 28), reflecting the general higher distribution of females among people with MS (Rotstein et al., 2018). The analysis of sociodemographic characteristics did not reveal any significant differences in age, EDSS scores and MS duration among the three groups (Table 2).

**TABLE 2:** Results of the homogeneity of groups according to Age, EDSS, and MS duration of the patients.

	Group			*p*
	II(*n* = 11)	MBI(*n* = 12)	CG(*n* = 12)	
Age (years)	43.00 ± [34.50–51.00]	44.00 ± [41.75–47.00]	44.50 ± [40.75–47.25]	0.96
EDSS (0 – 5.5)	4.00 ± [2.25–4.75]	3.50 ± [2.00–4.12]	3.50 ± [2.00–4.00]	0.84
MS duration (years)	12.00 ± [10.00–21.00]	17.50 ± [11.75–22.25]	17.50 ± [14.75–18.75]	0.57

*Values presented are median [interquartile range] for each characteristic. II = Implementation Intention, MBI = Mindfulness Based Intervention, CG = Control Group, EDSS = Expanded Disability Status Scale (score), MS = Multiple Sclerosis, P values assessed by Kruskal-Wallis test (ANOVA).*

Also, concerning the quality-of-life scale (EQ5D-3L), we didn’t find any significant differences in each of the groups examined.

The pre-intervention and post-intervention data for each group at week 1 and 8 is presented below in Table 3.

**TABLE 3:** Pre-intervention and Post-intervention values (Median ± interquartile range) for 6 minutes walking test (meter), MFIS and MSIS-29.

Variable	Mindfulness based intervention group				Implementation Intention group				Control group			
	Pre	Post	*p*	RBC	Pre	Post	*p*	RBC	Pre	Post	*p*	RBC
**6MWT** (meter)	429.50[264.75–476.25]	470.00[429.500–516.750]	<0.001	–0.974	435.00[287.50–477.50]	470.00[423.00–519.00]	0.002	–0.970	458.50[353.25–515.00]	506.50[475.75–540.25]	0.009	–0.833
**MFIS** (Cognitive component)	16.00[4.50–25.75]	11.00[7.50–19.25]	0.563	0.212	19.00[13.00–23.00]	14.00[9.00–17.50]	0.012	0.909	17.50[11.50–22.00]	14.00[8.75–19.25]	0.116	0.526
**MFIS** (physical component)	20.50[14.75–26.75]	16.00[10.50–20.50]	0.028	0.731	23.00[21.00–28.00]	19.00[12.00–22.00]	0.022	0.836	22.50[16.75–23.50]	16.50[14.25–18.50]	0.185	0.491
**MFIS** (Psychosocial component)	5.00[1.50–2.25]	2.50[1.75–4.00]	0.166	0.485	4.00[2.50–6.00]	3.00[1.50–4.00]	0.023	0.917	4.00[2.75–4.25]	3.00[1.75–3.25]	0.057	0.722
**MSIS-29_(**physical component) (0–100)	43.00[30.75–54.00]	36.50[22.75–39.500]	0.008	0.885	47.00[41.50–52.00]	39.00[32.50–42.50]	0.023	0.788	48.50[32.00–57.50]	37.00[31.00–41.25]	0.028	0.800
**MSIS-29_(**psychological component) (0–100)	25.00[13.75–33.75]	18.50[13.50–22.50]	0.107	0.538	25.00[20.00–26.00]	19.00[17.00–25.00]	0.264	0.394	22.00[17.75–26.25]	18.50[17.25–20.50]	0.286	0.379

*6MWT = 6 Min Walking Test, MFIS = Modified Fatigue Impact Scale, MSIS-29 = Multiple Sclerosis Impact Scale-29, P values assessed by Wilcoxon signed-rank t–test, RBC = Rank-Biserial Correlation.*

### Six-Minute Walk Test (6MWT)

Several within groups significant changes appeared between baseline (T1) and the end of the program (T2) for each examinate group. For the 6MWT there was a significant increase in the walked distance for the Mindfulness group (*p* < 0.001), Implementation Intention group (*p* = 0.002) and for the control group (*p* = 0.009).

### Multiple Sclerosis Impact Scale (MSIS-29)

The Wilcoxon signed rank test showed a statistically significant difference in change scores across the three groups for the MSIS-29 physical component (Table 3).

### Modified Fatigue Impact Scale (MFIS)

Concerning the MFIS score, we noted a significant decrease in the Implementation Intention group for the cognitive (*p* = 0.012), physical components (*p* = 0.022) and Psychosocial components (*p* = 0.023), a statistically significant decrease was revealed alone for the physical dimension in the MBI group (*p* = 0.028), whereas MFIS scores remain unchanged for the control group.

### MFIS-MVPA

When we associate the MFIS score with the duration of the practice of the physical activity within the sessions, we noted, in the II group, no correlation at T1 between MFIS (physical component) and time spent in moderate to-vigorous physical activity (MVPA) per day by accelerometry. In contrast, we found at T2 a statistically significant negative correlation (*r* = −0.745; *p* = 0.008) between MFIS (physical component) and MVPA. Also, at T2, we found a weak negative correlation (*r* = −0.237) with the psychological component of MFIS, but not statistically significant. This result may indicate that PA combined with a psychological intervention such as II decreases fatigability among people with MS and could improve PA practice (sustainability).

In MBI group, we noted no correlation in T1 and T2 between MFIS (physical component) and the time spent practicing PA (MVPA). Finally, in control group, we found at T2 a statistically significant positive correlation (*r* = 0.640; *Puncorrected* = 0.025) between MFIS (physical component) and MVPA, but this correlation did not survive when applying stringent adjustment for multiple comparisons across the correlations.

### Adhesion

The descriptive data of the adhesion to the psychological and motivational intervention follow up for each group eight weeks after randomization is presented in Table 4.

**TABLE 4:** Descriptive statistics of the adhesion to psychological and motivational support questionnaire percentile after eight weeks intervention.

	Implementation intention group (*n* = 11)	Mindfulness-based intervention group (*n* = 12)
Median	100%	53%
Minimum	88%	18%
Maximum	100%	82%
Interquartile range	6%	24.7%

## Discussion

The aim of this study was to assess the efficacy of a PA program compared to two types of psychological interventions – Mindfulness and Implementation Intention – delivered via internet, in reducing consequences of MS. Effects of practicing were unrelated to degree of impairment (i.e., EDSS level). Following the eight weeks intervention, the results of this study revealed that our PA program led to significant changes in the mobility of people with mild to moderate MS (EDSS < 5.5) in the three groups. The significant increase in distance covered in the 6-min walk test reflects these improvements. In their study, van Asch (2011) found that worsening locomotion was a major concern for both short and long-term MS patients (Heesen et al., 2008; van Asch, 2011). This indicates one of the most debilitating dysfunctions encountered by patients with MS (Motl et al., 2010). Mobility is impaired in up to 90% of people with MS (Zwibel and Smrtka, 2011), increasing the rates of falls and injuries (Nilsagård et al., 2009; Gunn et al., 2014) that may pose a threat to this population (Peterson et al., 2008; Cameron et al., 2011). Therefore, there is still a need to find strategies to increase mobility and maximize autonomy of these people, and the PA program has helped achieve this goal. This represents a significant extension of treatment options.

There was a significant decrease in the MSIS-29 physical component scores in the three groups, indicating a decrease of the impact of MS on patient’s day-to-day life. A broad range of studies have assessed the effects of exercise and PA on MSIS-29. Results revealed improvement in MSIS-29 sub-scales relative to controls (Tollár et al., 2020). Our findings are in line with the results found in the literature. However, our study provided evidence that a PA program combined with mindfulness and motivational interventions may be more promising and present more advantages in terms of amelioration of MS symptoms. Although our analysis did not reveal a significant change in the psychological component of MSIS-29, we noticed a decrease (about 20%) in the average scores, indicating that a longer practice over time may lead to significant changes in this component.

A contribution of this study was to observe that an Implementation Intention carried out in conjunction with the PA program resulted in significant reductions in Modified Fatigue Impact Scale (MFIS) for all factor scores (physical, cognitive, psychosocial) reviewed by some authors to be three important sub-scales in people with MS (D’Souza, 2016). It might be that fatigue had less impact on patients’ lives for the Implementation Intention group during the past 8 weeks. Finally, when it comes to the likelihood and speed of performance, the Implementation Intention intervention (if-then plan) seems to be more effective than simple intentions (“I intend to reach *!”). In fact, specific plans for when, where, and how to initiate behavior and solve difficult tasks almost automatically generate behaviors that help individuals translate their intentions into behaviors (Schwarzer et al., 2008). More specifically, the mental representation of the action becomes highly activated and thus more easily accessible (Gollwitzer and Sheeran, 2006). In contrast, Mindfulness Intervention associated with the PA program produced significant changes only in the physical component. There are many benefits of Mindfulness interventions mentioning the prevention of deteriorating of working memory during stressful periods (Jha et al., 2010).

In people with MS, impairments in working memory appears at the early stage of the disease (Pelosi et al., 1997). In addition, Mindfulness practice can elicit improved visuospatial processing efficiency (Kozhevnikov et al., 2009), enhanced attention (Brefczynski-Lewis et al., 2007) and reduce mind wandering caused by a reduced activation of the default mode network (Mrazek et al., 2013). According to literature (Wayne and Kaptchuk, 2008), mindfulness-related practices (e.g., tai chi) may more commonly engender the rehabilitation and prevention benefits after many years of practice. Thus, larger sample sizes and longer session durations (> 8 weeks) may lead to the discovery of statistical significance improvement of cognitive and sociopsychological factors. In addition, it is important to adapt Mindfulness Interventions to chronic condition such as MS since it may be challenging to find 1 h per day and focus. Some other forms of Mindfulness interventions such as Loving Kindness Meditation, a rich behavioral intervention easier to practice and implement (Galante, 2014) may be more suitable for people with MS. Moreover, the scores of the three components were unchanged for the control group who did not receive any psychological Intervention. Results indicate that 8 weeks Physical activity program by itself was not sufficient to produce modifications relating to the impact of fatigue in the patient’s life.

Fatigue is one of the most common symptoms, and studies conducted in patients with MS confirm that about 50–60% of them suffer from this most troublesome symptom (Costello and Harris, 2003; Trojan et al., 2007). Fatigue is defined as “a subjective lack of physical or mental energy that is perceived by the individual or caregiver to interfere with activities of daily living” (Kos et al., 2006). It can be caused by peripheral neuropathy (e.g., impaired neurotransmission) (Fisk et al., 1994). In addition to its negative effects on cognitive functions, decreasing individuals’ attention and concentration, fatigue may limit people with MS activities of daily living (Mollaòlu and Üstün, 2009) and pose a serious barrier to rehabilitation (Michael, 2002). Furthermore, fatigue may contribute to decline in cognitive performance (Krupp and Elkins, 2000). For the above-mentioned reasons, elaborating instant and effective strategies to reduce fatigue in people with MS must be a priority.

When it comes to MVPA, numerous studies provided clear evidence that people with MS are less physically active than healthy subjects (Motl et al., 2005; Sandroff et al., 2012). People with MS do not engage in sufficient amounts of MVPA recommended by the American College of Sports Medicine. This is a concern, since studies have demonstrated that people with MS only accrue health benefits when practicing MVPA (Garber et al., 2011). Klaren et al. (2015) exhibited the existence of a reconcilable link between MVPA (minutes/day), but there is no such case regarding light physical activity or sedentary behavior, and whole brain gray/white matter and deep gray matter structures including hippocampus, thalamus, caudate, putamen, and pallidum volumes. Those with MS who had high levels of MPVA (minutes/day) had also higher amounts of white matter and deep gray matter structures (Klaren et al., 2015). The deterioration of these subcortical structures occurs in people with MS (Hulst and Geurts, 2011; Gh Popescu and Lucchinetti, 2012). Such deterioration was associated with disability status (Shiee et al., 2012) and cognitive dysfunction (Rao et al., 1989; Sanfilipo et al., 2006) in people with MS. On the other hand, literature in this area have identified MS-related fatigue as the main cause of this restriction to participation in PA in MS (de Groot et al., 2005, 2008). Here, we provided data indicating that II combined with a PA program decreases fatigability among people with MS and that fatigue was strongly negatively associated to the time spent practicing MVPA in people with mild to moderate MS for the II group which was not the case with the control group. This result may indicate that PA combined with a psychological intervention such as II decreases fatigability among people with MS which allows the subject to spend more time in MVPA to better benefit from the multiple advantages previously mentioned in order to improve his condition.

## Strengths and Limitations

This is the first with randomized controlled trial (RCT) assessing Mindfulness Interventions associated with physical activity with people with MS, a clinical population difficult to recruit due to the numerous impairments produced by the disease. There are no other clinical trials known to the authors that have reported the effects of combining PA with mindfulness and motivational interventions in people with MS. The results of this study show that such interventions are usable for people with MS, as they may improve mobility and other measures impaired by disease (e.g., fatigue) achievable in a very short period, and at a very low cost.

Nevertheless, this research has several limitations. Firstly, a small sample size and the lack of follow-up data to check the stability of the results over time. Additional studies with larger sample size and follow-up assessments are needed to confirm these initial findings, in order to add to the existing literature on combined interventions among people with MS. Finally, we emphasized the lack of follow-up of the Mindfulness training proper conduct. Unfortunately, the only information that was at our disposal is that patients were connected to the application. First, we do not know if they were really listening to the recordings, because being connected does not give reliable information, and if so, whether they listened to all the recordings or whether they were able to focus their attention on the instructions. Indeed, the cognitive impairment associated with the MS condition can make it really challenging to follow this type of Mindfulness training. Our application was not sufficiently developed to provide us all this type of information. In addition, for this pilot study, six pre-recorded sessions of 10 min each delivered via an internet application were used, six days per week. The training program was diversified, composed of a large number of situations aiming at developing one’s proprioception, situations aiming at developing balance and posture and finally situations aiming at developing awareness and rhythm while walking or breathing. However, 6 h home training per week seems to be a long and challenging activity for people with MS. Some other forms of Positive psychology interventions such as Loving Kindness Meditation may be more adapted to this population since there is a dose effect and equivalent result in a much shorter period (10 min per day), (Kirby and Laczko, 2017). Therefore, the procedure used for our MBI method should be reviewed to make it less burdensome for the patient. Additionally, in order to increase motivation, further studies may consider using live video sessions shared by several patients instead of using pre-recorded sessions. We also propose the use of daily reminders and boosters to encourage him to participate in the session. Finally, we also indicate that the actimetry was used “blind” for the patient who did not have access to his amount of daily physical activity. It would be better, in a future study, to inform the patients of the amount of daily physical activity and enable the set of daily goals. Finally, the disposition to full consciousness was not taken into account in this study which explains some of the results in the MBI group which would have made it possible to obtain results larger than the only physical component.

## Conclusion and Perspectives

This randomized clinical trial clearly indicates that a PA program tailored to people with MS associated with psychological interventions could be beneficial for people with MS across a broad range of variables. This study reveals that PA could improve mobility and MSIS-29 physical scores in people with mild and moderate MS. Moreover, PA accompanied with Mindfulness Intervention seems to be effective in improving mobility and reducing the impact of the disease in patients’ life. Nevertheless, the mechanisms by which Mindfulness achieves these benefits remain unclear (Grossman et al., 2010). On the other hand, Implementation Intention associated with PA is also effective in enhancing mobility, but more beneficial in decreasing fatigue among people with MS. Another conclusion that we could draw from this study is that II was an activity that is easy to perform, and its practice does not require a long period of time, this was reflected by the percentages of the adhesion to the II questionnaire which were very high within the group. Our study showed that each of the two psychological interventions associated with a PA program has beneficial but different effects among people with mild to moderate MS. Clearly, results revealed that each combination induced a reduction of the specific disease symptoms.

We further propose two ways for improving this research in people with MS as an extension of this work. In this study, we did not seek to understand the mechanisms by which these psychological interventions achieve benefits. Functional magnetic resonance imaging (fMRI) may be a good way to identify the mechanisms by characterizing the brain regions and functional connectivity involved in these psychological states to shed light on its mechanisms of action. A neuroplasticity induced by a Mindfulness intervention has been observed in brain structures and functions. Gotink et al. (2016) showed, using a Mindfulness intervention, an increased connectivity and volume of the prefrontal cortex related to meta-awareness, emotion regulation (anterior cingulate cortex), body awareness (insula) and memory (hippocampus). The brain regions which were influenced by Mindfulness may induce changes that are of significance to emotional modulation and that might be pertinent to improve cognitive functions in people with MS (Willekens et al., 2018).

This is the first study to assess the efficacy of a positive psychology intervention associated with physical activity in people with MS. MS is a severe condition with multiple impairments (cognitive, chronic fatigue, mood disorders) and psychological interventions may be challenging for people with MS and need adjustments. A recent study assessed the feasibility and acceptability of a 5-week group positive psychology intervention for patients with MS. The results are promising, as they revealed a significant improvement in fatigue and depression after the intervention (*P* = 0.016 and 0.049, respectively). In addition, they showed the feasibility and acceptability of this type of intervention among MS population (Leclaire et al., 2018). However, the study was not randomized nor controlled, with a small sample (*n* = 17). A very recent RCT (Freedman et al., 2021) examined the feasibility, acceptability, and impact of a 5-week, telephone-delivered positive psychology (PP) intervention for individuals with Multiple Sclerosis (MS). The PP intervention was associated with significantly greater increases (*p* < 0.05) in positive affect, optimism, state and trait anxiety, general health, and resilience in the intervention group versus the control group (waitlist control). Moreover, the results were stable in time, since half of the PP participants maintained at least 50% of the improvement at 10 weeks. Randomized controlled trials (RCT) are thus necessary to further explore the effectiveness of the positive psychology interventions with people with MS.

We believe that positive psychology is a very promising method of psychological intervention for people with MS as they propose attractive and easy to implement strategies and focus on positive aspects. Studies assessing the effectiveness of the PPIs have shown a high level of heterogeneity: there is a wide variability in effect size across PPIs. It is therefore necessary to examine the specificities of the different interventions included in the PPIs (Csillik, 2015). Classical Mindfulness Interventions address both positive and negative aspects of human functioning. In recent years, Positive Psychology Interventions (PPIs) that integrate mindfulness elements have shown promising outcomes (Lomas and Ivtzan, 2016). Still, there is a lack of clarity in the implementation of Mindfulness-Based Positive Psychology Interventions (MPIs) and their impact on positive human functioning. A recent review aimed to find and analyze the mindfulness-based interventions from the existing literature which have also shown potentials to be a positive psychology intervention (Allen et al., 2021). The outcomes point out the positive potentials of MBIs and the endless possibilities of empirical studies on the application of MPIs and, emphasizes the need of future studies paying attention to positive outcomes when measuring the effects of Mindfulness Based Interventions. In line with these recommendations, we aim to further assess the effects of MBIs adapted to people with MS on other positive outcomes that are more sensitive to change such as eudemonic and hedonic well-being in addition to the quality-of-life measures that are less sensitive to change and to not specifically focus on the people’s own feelings and perceptions of their levels of happiness and well-being.

## Data Availability Statement

The raw data supporting the conclusions of this article will be made available by the authors, without undue reservation.

## Ethics Statement

The studies involving human participants were reviewed and approved by Ile de France XI (ID: 2016-A00537-44) authorized from the National Agency for the Safety of Medicines and Health (ASNM) and registered under the identification number ID: NCT03785483. The patients/participants provided their written informed consent to participate in this study.

## Author Contributions

ED, JS, ET, AC, M-CG, OH, DB, and GDM: organization and execution of the research project and review and critique. ED, JS, and ET: data collection. GDM, ET, and AC: statistical analysis and writing manuscript. All authors contributed to the article and approved the submitted version.

## Conflict of Interest

The authors declare that the research was conducted in the absence of any commercial or financial relationships that could be construed as a potential conflict of interest.

## Publisher’s Note

All claims expressed in this article are solely those of the authors and do not necessarily represent those of their affiliated organizations, or those of the publisher, the editors and the reviewers. Any product that may be evaluated in this article, or claim that may be made by its manufacturer, is not guaranteed or endorsed by the publisher.

## Footnotes

1
https://www.tailorbuilder.com/en/about.html
